# Looming Effects on Attentional Modulation of Prepulse Inhibition Paradigm

**DOI:** 10.3389/fpsyg.2021.740363

**Published:** 2021-11-15

**Authors:** Zhemeng Wu, Xiaohan Bao, Lei Liu, Liang Li

**Affiliations:** School of Psychological and Cognitive Sciences, Beijing Key Laboratory of Behavior and Mental Health, Peking University, Beijing, China

**Keywords:** auditory looming bias, prepulse inhibition, attention, isolation rearing, rats

## Abstract

In a hazardous environment, it is fundamentally important to successfully evaluate the motion of sounds. Previous studies demonstrated “auditory looming bias” in both macaques and humans, as looming sounds that increased in intensity were processed preferentially by the brain. In this study on rats, we used a prepulse inhibition (PPI) of the acoustic startle response paradigm to investigate whether auditory looming sound with intrinsic warning value could draw attention of the animals and dampen the startle reflex caused by the startling noise. We showed looming sound with a duration of 120 ms enhanced PPI compared with receding sound with the same duration; however, when both sound types were at shorter duration/higher change rate (i.e., 30 ms) or longer duration/lower rate (i.e., more than 160 ms), there was no PPI difference. This indicates that looming sound–induced PPI enhancement was duration dependent. We further showed that isolation rearing impaired the abilities of animals to differentiate looming and receding prepulse stimuli, although it did not abolish their discrimination between looming and stationary prepulse stimuli. This suggests that isolation rearing compromised their assessment of potential threats from approaching objects and receding objects.

## Introduction

The detection of an approaching object is fundamentally important to the survival of an organism. For instance, the decision by an animal in the wild to forage for food requires an evaluation of predation by assessing their approaching behavior (e.g., faster pace) and their associated probabilities of occurrence and magnitudes. In auditory field, looming sounds with rising intensity and receding sounds with falling intensity are primary cues to aid in judging the motion of objects (Seifritz et al., 2002; Hall and Moore, 2003; Baumgartner et al., 2017). The phenomenon of looming sounds containing an intrinsic and unconditioned warning value therefore being more salient than receding sounds, is termed as “auditory looming bias” (Seifritz et al., 2002; Hall and Moore, 2003; Maier et al., 2004; Baumgartner et al., 2017; Glatz and Chuang, 2019; Bidelman and Myers, 2020). Previous literature reveals that sound spectrum affects the perception of auditory looming bias. Compared with looming white noise with equal increasing intensity, complex pure tones (Neuhoff, 1998, 2001) or harmonic tones (Deneux et al., 2016) elicited stronger auditory looming bias. For example, macaques were more attracted to the approaching harmonic tones by orienting them to longer time than receding tones (Ghazanfar et al., 2002).

Prepulse inhibition (PPI) of the acoustic startle response paradigm is the suppression of the startle reflex when an intense startling stimulus is preceded by a weaker sensory stimulus (the prepulse) (Fendt et al., 2001; Du et al., 2009; Li, 2009; Li et al., 2009). Graham (1975) proposed a “protection of processing” theory for justifying the function of PPI: the weaker prepulse preceding the startling noise triggers a gating mechanism that dampens the disruptive effects caused by the startling noise (Graham, 1975). Therefore PPI has been recognized as an operational cross-species measure of sensorimotor gating mechanism to help humans and animals adapt to the complex environment (Swerdlow et al., 2001, 2006). The “protection of processing” theory of PPI (Graham, 1975) makes PPI a good behavioral paradigm for studying how the salient value of auditory looming sounds as a prepulse would protect the organism from interference by disruptive stimuli. In this study, we used PPI of the acoustic startle response paradigm to investigate the effects of looming sounds on sensorimotor gating in rats.

Previous literature shows that emotional or spatial attention to the prepulse can enhance PPI of the acoustic startle response. In rats, when a prepulse is paired with the foot shock and becomes emotionally salient, PPI induced by this fear-conditioned prepulse is enhanced (Du et al., 2009, 2010, 2011; Li, 2009; Li et al., 2009). Additionally, PPI can be further enhanced by a spatially separated conditioned prepulse from background noise masker than a spatially co-located prepulse with the masker (Du et al., 2009, 2010, 2011; Li, 2009; Li et al., 2009), which illustrates that spatial attention to the prepulse enhances PPI. Both the emotional fear conditioning–induced PPI enhancement and perceptual spatial separation–induced PPI enhancement reveal that PPI can be modulated by attention (Li et al., 2009). These attentional enhancements of PPI are achieved by acquiring salient valence of prepulse (e.g., fear conditioning), and it is of interest to know whether a prepulse with natural and intrinsic salience (e.g., looming sounds) can also draw the attention of rats, therefore enhancing the PPI. The main aim of this study was to investigate how the auditory looming sound as a prepulse affects PPI compared with the receding sound.

Looming sounds are perceived as spanning a longer duration compared with receding sounds with the same presentation duration (Schlauch et al., 2001; Grassi and Darwin, 2006; DiGiovanni and Schlauch, 2007; Grassi, 2010). This subjective different temporal judgment between looming and receding sounds indicates that looming sounds are attended for longer duration than receding sounds (Glatz and Chuang, 2019). As the duration of prepulse has an impact on PPI (Swerdlow et al., 2001; Li et al., 2009), in our study, we manipulated the duration/rate of both looming and receding sounds as prepulse and kept the total amount of intensity change the same across the two prepulse stimuli (i.e., 50 dB SPL), to investigate whether the auditory looming effects on PPI are duration/rate dependent. As the total amount of intensity change was equal to the duration multiplied by the change rate of prepulse, in our study, change of duration also caused change of intensity rate for looming and receding sounds.

Isolation rearing starting from early life is one of the commonly used animal models, to mimic the behavioral and cognitive impairments in many mental disorders, such as schizophrenia (Li et al., 2009; Wu et al., 2018), depression and anxiety (Thorsell et al., 2006; Mosaferi et al., 2015; Ma et al., 2017; Harvey et al., 2019), attention-deficit hyperactivity disorder (ADHD) (Yates et al., 2012; Ouchi et al., 2013), and other general early-life stress effects upon the brain and behavior during adulthood (Weiss et al., 2000, 2004). Among the cognitive/behavioral deficits caused by isolation rearing, attentional impairment is found in several behavioral paradigms (Wu et al., 2018). For example, it has been found that isolation rearing abolished both emotional and spatial attentional enhancements of PPI (Du et al., 2009, 2010; Li et al., 2009; Lim et al., 2012; Wu et al., 2016), declined attentional processing to novelty (Atmore et al., 2020), and impaired attentional set shifting (McLean et al., 2010). As looming sounds contain an intrinsic salient value for organism to avoid danger, it is of interest to investigate how isolation rearing would preserve or abolish this intrinsic attention to approaching objects. The second aim of this study was to investigate how isolation rearing affects auditory looming effects on attentional modulation of PPI. As previous literature documented an attentional deficit caused by isolation rearing (Du et al., 2009, 2010; Li et al., 2009; McLean et al., 2010; Lim et al., 2012; Wu et al., 2016, 2018; Atmore et al., 2020), we hypothesized that isolation rearing might impair the abilities of animals to differentiate looming and receding sounds; therefore no PPI difference between the two prepulse stimuli would be observed.

## Materials and Methods

### Animals

Twelve Sprague–Dawley rats participated in Experiment 1. Another eighteen Sprague–Dawley pups were involved in Experiment 2. All the rats and the pregnant female rats were purchased from the Vital-River Experimental Animals Technology Ltd., Beijing, China. They were transported in a special vehicle to our laboratory in cages of five to six animals each. All animal training and experimental procedures were performed in accordance with the guidelines of the Beijing Laboratory Animal Center, and the Policies on the Use of Animals and Humans in Neuroscience Research approved by the Society for Neuroscience (2006).

The twelve adult Sprague–Dawley rats in Experiment 1 were socially housed and tested after approximately 2 months of their arrival to the laboratory. The eighteen pups remained in the litter along with their lactating mothers until weaning on postnatal day (PND) 21. After weaning, each of the pups was randomly assigned to either the social rearing group (reared in pairs, *N* = 10) or the isolation rearing group (reared individually, *N* = 8) for 8 weeks. The pups assigned to either the social or isolation rearing group were tested in Experiment 2. The eighteen pups in Experiment 2 were tested on their PND 77. Each individual rat (isolation-reared) and three individual rats (socially reared) were housed in a single transparent plastic cage (48 × 30 × 18 cm, the cage size was the same in socially reared and isolation-reared rats), under a temperature of 24 ± 2°C and a 12-h light/dark cycle with food and water freely available.

### Stimuli and Apparatus

**FIGURE 1:**
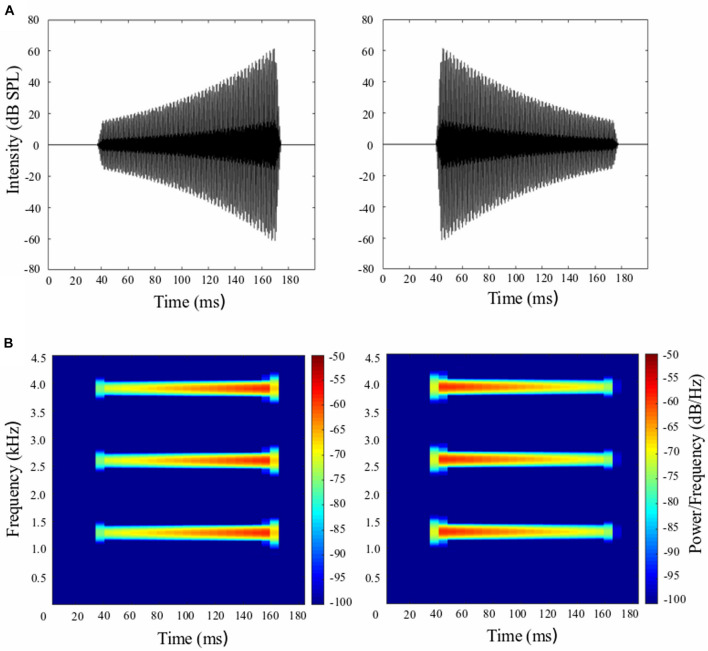
Rising- and falling-intensity harmonic tone stimuli used in the prepulse inhibition (PPI) paradigm. **(A)** The waveform of looming prepulse with rising intensity from 17 to 67 dB SPL (left panel) and receding prepulse with falling intensity from 67 to 17 dB SPL (right panel) with a duration of 120 ms. **(B)** The frequency spectrum for looming prepulse (left panel) and receding prepulse (right panel).

The whole-body startle reflex of rat was induced by an intense 10-ms broadband noise burst (0–10 kHz, 100-dB SPL) delivered by a loudspeaker above the rat’s head. The electrical voltage signals were sampled at 16 kHz and collected for 500 ms. In a single trial, as the startling stimulus could reliably induce a distinct waveform complex of the startle response (Zou et al., 2007; Du et al., 2011), the startle response was digitized and measured as the peak-to-peak amplitude between the primary peak component and the subsequent peak component. The prepulse stimulus, which ended 50 ms prior to the startling noise, was a 30, 120, 160, or 200-ms three-harmonic tone complex (1.3, 2.6, 3.9 kHz) with rising intensity (i.e., looming prepulse) and falling intensity (i.e., receding prepulse) (Figure 1). The sound level of looming prepulse rose from 17 to 67 dB SPL; and the sound level of receding prepulse dropped from 67 to 17 dB SPL. The stationary prepulse was the same three-harmonic tone complex with a stable intensity of 67 dB SPL. We controlled the amount of intensity change the same for looming and receding prepulse stimuli (i.e., 50 dB SPL) and changed the duration, therefore the corresponding rate for 30, 120, 160, and 200 ms prepulse could be calculated as (67–17)/30 = 1.67 dB SPL/ms; (67–17)/120 = 0.42 dB SPL/ms; (67–17)/160 = 0.31 dB SPL/ms; and (67–17)/200 = 0.025 dB SPL/ms. We could see shorter duration was accompanied with higher change rate and *vice versa*. The root mean square (RMS) level of looming and receding prepulses were identical: the RMS value was 7.0, 24.5, 32.3, and 40.1 for durations of 30, 120, 160, and 200 ms, respectively. This indicates that the energy was the same for all prepulses at a given duration. The Sprague–Dawley albino rats have the greatest hearing sensitivity, which is between 8 and 38 kHz. The best hearing points are 8 kHz and 32–38 kHz. Between 8 and 32 kHz, the sensitivity levels slightly decline (Kelly and Masterton, 1976; Borg, 1982; Heffner et al., 1994). Although the prepulse stimuli used in our study are not within the range of the highest hearing sensitivity, the rat’s hearing threshold for 1–2 kHz tones is approximately 25 dB SPL and for 3–4 kHz tones is approximately 15 dB SPL (Kelly and Masterton, 1976; Borg, 1982; Heffner et al., 1994). Thus, our rats could detect the stationary prepulse stimuli at 67 dB SPL, looming and receding prepulse stimuli at 3.9 kHz above 15 dB SPL (i.e., within the range of 17–67 dB SPL), and at 1.3 and 2.6 kHz above 25 dB SPL. The 17dB SPL is more likely not loud enough to detect the 1.3 and 2.6 kHz tone of the three-harmonic tone complex. This is one of the weaknesses of our study when choosing the frequency and intensity of three-harmonic tone complex as looming and receding prepulse stimuli. The prepulse was delivered by each of the two horizontal and spatially separated loudspeakers, which were placed horizontally in the frontal field with a 100° separation angle and 52 cm away from the rat’s head position. All the sound stimuli were digitally generated by Adobe Audition software and converted by a custom-developed sound delivery system (National Key Laboratory on Machine Perception, Peking University). Calibration of sound intensity was conducted with a Larson Davis Audiometer Calibration and Electro-acoustic Testing System (AUDit & System 824, Larson Davis, Depew, NY, United States).

### Testing Procedures

On the first three successive days, the rat was placed inside a restraining cage, whose dimensions matched the size of the rat body so that the rat could not reorient its head and body positions, and the rat was exposed to the broadband noise (60 dB SPL) delivered for 30 min. The broadband noise was continuously presented by each of the two horizontal loudspeakers. Neither the prepulse nor the startling noise was presented during the acclimation. This procedure was to adapt the rat to the restraining cage and testing chamber. The enclosure was wiped down with ethanol solution after each animal.

On the fourth day (test day), the rat was tested in the same environment embedded with the background noise. The rat received 10 presentations of startling stimulus without prepulse presentation for the first 5 min. In Experiment 1, the testing block included 45 trials: 5 trials containing startling noise alone, 20 trials containing a looming prepulse with rising intensity preceding startling noise (with 5 trials per each prepulse duration), and 20 trials containing another receding prepulse with falling intensity preceding startling noise (with 5 trials per each prepulse duration). In Experiment 2, the duration of prepulse was fixated (i.e., 120 ms) based on the result of Experiment 1, the testing block included 20 trials: 5 trials containing startling noise alone, 5 trials containing a looming prepulse preceding startling noise, and 5 trials containing a stationary prepulse preceding startling noise, and 5 trials containing a receding prepulse preceding startling noise. The three types of trials within a testing block were presented in a random order, and this order of the presentation was randomized across rats. The randomization of the presentation order and rat order was created using a “RAND” formula in Excel. The inter-stimulus onset interval between a prepulse and the startling stimulus was fixated at 100 ms. The interval between each trial varied between 25 and 35 s (mean = 30 s).

### Data Analysis

The startle response was measured as the peak-to-peak amplitude between the primary peak component (latency mainly between 15 and 20 ms) and the subsequent peak component (latency mainly between 20 and 25 ms), and were baseline corrected. The startle responses were reliable across all the rats. All the trials, including startling noise-only trials and prepulse trials, were included in the analysis. The value of PPI of the acoustic startle response was calculated with the below formula:


$$PPI\left(\%\right)=\frac{\begin{array}{c} (amplitudetostartlingnoisealone-amplitude\\ tostartlingnoiseprecededbyprepulse) \end{array}}{a\,m\,p\,l\,i\,t\,u\,d\,e\,t\,o\,s\,t\,a\,r\,t\,l\,i\,n\,g\,n\,o\,i\,s\,e\,a\,l\,o\,n\,e}\times 100\%$$

In Experiment 1, a two-way repeated-measures ANOVA with the factors prepulse duration (four levels) and prepulse type (two levels) was conducted. In Experiment 2, a two-way mixed-design ANOVA with the factors rearing conditions (independent factor, two levels) and prepulse type (dependent factor, three levels) was conducted. All the statistical analyses were performed using SPSS 15.0 software. The null hypothesis rejection level was set at 0.05. The data were presented using raincloud plots (Allen et al., 2019).

## Results

### Experiment 1: Looming Effects on Attentional Modulation of Prepulse Inhibition at Different Prepulse Durations in Socially Reared Rats

**FIGURE 2:**
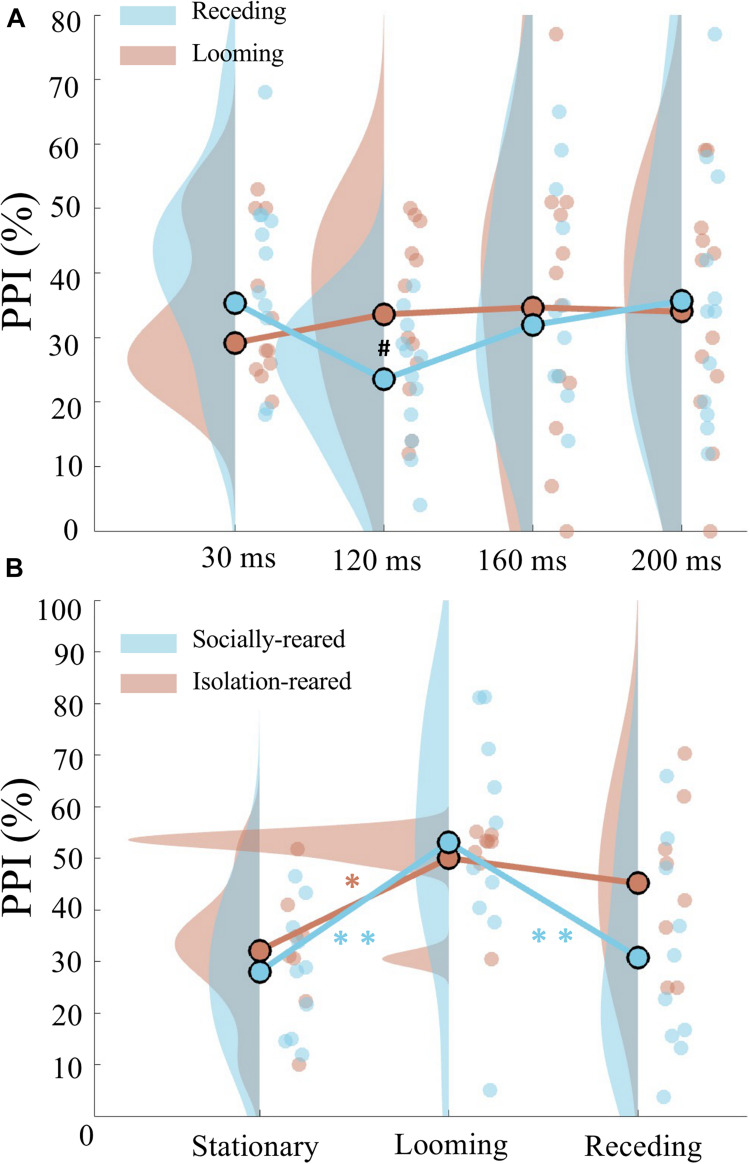
Prepulse duration and rearing type on looming-induced attentional modulation of PPI. **(A)** Raincloud plots of PPI induced by looming prepulse (red) and receding prepulse (blue) when the prepulse duration was 30, 120, 160, and 200 ms. Note that a marginal significant difference of PPI between the looming and receding prepulses was observed when both the prepulses were 120 ms. **(B)** Different modulated PPI by two rearing types. Not that in socially reared rats (blue), PPI was significantly increased by looming prepulse than PPI induced by stationary and receding prepulse; in isolation-reared rats (red), PPI was only increased by looming prepulse than PPI induced by stationary prepulse. No difference of PPI between looming and receding prepulses was found. Circles are individual data; circles with black edge color show the average of group data; distributions show probability density function of data points. ^∗^Indicates *p* < 0.05, ^∗∗^indicates *p* < 0.01, and ^#^indicates *p* < 0.1.

We found a difference of PPI induced by looming prepulse with rising intensity and receding prepulse with falling intensity that depended on the duration of prepulse (Figure 2A). A two-way repeated measures ANOVA was conducted on the PPI data, with prepulse type (2 levels: looming and receding) and prepulse duration (4 levels: 30, 120, 160, and 200 ms) as the within-subject factor. We found a marginal significant quadratic trend interaction for prepulse duration (*F*_(__1_,_11__)_ = 3.441, *p* = 0.091, η*^2^* = 0.238). The pairwise *t*-tests further showed that PPI elicited by looming prepulse was marginally significantly larger than the PPI induced by receding prepulse when the duration of prepulse was 120 ms (*t*_(__11__)_ = 2.715, *p* = 0.020, Bonferroni corrected *p* = 0.080). However, no significant difference of PPI occurred between looming and receding prepulse at 30 ms (*t*_(__11__)_ = −1.764, *p* = 0.105, Bonferroni corrected *p* = 0.420), 160 ms (*t*_(__11__)_ = 0.296, *p* = 0.773, Bonferroni corrected *p* = 1.000), and 200 ms (*t*_(__11__)_ = −0.387, *p* = 0.706, Bonferroni corrected *p* = 1.000).

### Experiment 2: Different Looming Effects on Attentional Modulation of Prepulse Inhibition in Socially Reared and Isolation-Reared Rats

In Experiment 1, the difference of looming-induced PPI enhancement occurred when the duration of prepulse was 120 ms, thus we chose 120 ms prepulse and further investigated whether the looming-induced PPI enhancement would be different between socially reared and isolation-reared rats in Experiment 2.

A two-way mixed-design repeated measures ANOVA was conducted on the PPI data, with prepulse type (three levels: stationary, looming, and receding) as the within-subject factor and group (two levels: socially rearing, isolation rearing) as the between-subject factor. A significant main effect of prepulse type (*F*_(__2_,_32__)_ = 16.872, *p* = 0.00001, η*^2^* = 0.513) was found, in addition to a significant linear trend interaction between prepulse type and group (*F*_(__1_,_16__)_ = 5.787, *p* = 0.029, η*^2^* = 0.266). The latter prompted further analysis on the two animal groups. In socially reared rats, repeated measures ANOVA showed a main effect of prepulse type (*F*_(__2_,_18__)_ = 16.416, *p* = 0.000088, η*^2^* = 0.646). The *post hoc* pairwise *t*-tests with Bonferroni correction revealed a larger PPI induced by looming prepulse than stationary prepulse (*p* = 0.002) and a larger PPI induced by looming prepulse than receding prepulse (*p* = 0.004). In isolation-reared rats, another repeated measures ANOVA showed a main effect of prepulse type (*F*_(__2_,_14__)_ = 5.030, *p* = 0.023, η*^2^* = 0.418), which prompted further scrutiny by *post hoc* pairwise *t*-tests with Bonferroni correction. These revealed a significant larger PPI induced by looming prepulse compared with PPI induced by stationary prepulse (*p* = 0.034), although no significant difference of PPI between the looming and receding prepulses was found (*p* = 1.000) (Figure 2B).

## Discussion

The results of our study demonstrated that the looming sounds with an adequate duration (i.e., 120 ms) induced PPI enhancement compared with receding sounds with the same duration, suggesting that approaching sounds serve an intrinsic warning cue for individuals to dampen the startle response elicited by a sudden and interfering stimuli. The “auditory looming bias” shown in previous literature (Neuhoff, 2001; Ghazanfar et al., 2002; Seifritz et al., 2002; Hall and Moore, 2003; Maier et al., 2004; Maier and Ghazanfar, 2007; Grassi, 2010; Baumgartner et al., 2017; Glatz and Chuang, 2019; Bidelman and Myers, 2020) was also found in PPI of the acoustic startle response paradigm in rats. We further showed that this looming effect–induced PPI enhancement was time or rate dependent. When the duration of looming prepulse was either too short (e.g., 30 ms, a faster rate) or too long (e.g., longer than 160 ms, a slower rate), PPI enhancements induced by the looming sounds disappeared for the fact that there was no difference of PPI elicited by the looming and receding sounds. This illustrates that an adequate processing time of approaching objects is needed to capture the attention of the rats and therefore enhanced PPI. It is also noted that recency effect of looming sounds could not explain our results. If rats differentiated looming and stationary prepulse based on recency effect, we should have found no difference of PPI between stationary (67dB SPL) and looming prepulse (the last tail intensity was 67 dB SPL). However, we found a significant difference between these two prepulse stimuli in both the animal groups. Previous literature documented various duration of approaching sounds–induced auditory looming bias in animals and humans: macaques demonstrated behavioral bias for looming sounds at 750 ms (Ghazanfar et al., 2002) and their neural discharge rate for rising and falling intensity sounds differed at a shorter duration (i.e., 25 ms) (Lu et al., 2001); humans judged longer for approaching than receding sounds when they were in the duration of 200 ms (Schlauch et al., 2001), or as long as 1,000 ms (Grassi and Darwin, 2006). How the temporal information is maintained in looming sounds and how the duration of looming sound affects the auditory looming bias are of interest for future research to advance.

The cognitive mechanism of different perceptions of looming and receding sounds has been studied in previous literature. One of the mechanisms is that looming sounds induced a loudness bias whereby its highest intensity of looming sounds was perceived in the end, therefore looming sounds were perceived louder than receding sounds and more easily captured attention (Neuhoff, 2001; Susini et al., 2010; Aumond et al., 2017). Another explanation is that looming sounds contained the first unattenuated segment which received the highest weight and therefore captured attention, while receding sounds had their first fade-in segments which were ignored (Oberfeld and Plank, 2011). For example, the decay portions or the tails of receding sounds were easy to be ignored, especially in a reverberant environment (e.g., the background broadband noise in our experiment), thus the tails may not be considered a meaningful part for the judgment of sound motion (DiGiovanni and Schlauch, 2007).

The neural mechanisms of perceiving looming and receding sounds differed. In animals, auditory looming sounds activated primary auditory cortex stronger than receding sounds in marmosets (Lu et al., 2001) and macaques (Maier and Ghazanfar, 2007). In humans, neuroimaging evidence showed that compared with receding sounds, looming sounds activated a broader neural network subserving motion perception, including the motor and premotor cortices and the cerebellum; and subserving attention, compromising the superior temporal sulci, the middle temporal gyri, and the temporoparietal junction (Seifritz et al., 2002). Another magnetoencephalography study in humans also confirmed that rising intensity complex sounds induced a stronger activation in bilateral inferior temporal gyrus and right temporoparietal junction compared with falling intensity complex (Bach et al., 2015). Taken together, looming sounds were more salient than receding sounds and involved more activations in temporal and frontal areas in animals and humans.

The neural circuitry mediating PPI is mostly located in the midbrain, comprising inferior colliculus (Li et al., 1998a,b, 2009), deeper layers, and intermediate layers of the superior colliculus (Fendt et al., 2001; Li et al., 2009), and primary auditory cortex (Du et al., 2011). PPI can be enhanced when the prepulse is endowed with emotional attention. For example, when the salient valence of prepulse was acquired by pairing it with foot shock, it has been found that this emotional attentional modulation of PPI relied on the lateral amygdala of rats (Du et al., 2011). Furthermore, this fear-conditioned prepulse induced larger PPI when this prepulse was spatially separated from background noise masker than the same prepulse was co-located with the masker. This spatial attentional modulation of PPI depended on the posterior parietal cortex of the rats (Du et al., 2011). One of the potential areas in mediating the approaching sound–induced PPI enhancement may be the primary auditory cortex of rats, as it was found to be involved in both the neural circuit of regulating PPI (Li et al., 2009; Du et al., 2011) and looming sound perception (Lu et al., 2001; Maier and Ghazanfar, 2007). Compared with receding prepulse, the approaching or looming prepulse contained an intrinsic salient value, which could draw attention to it, therefore enhanced PPI.

Besides primary auditory cortex, prefrontal cortex (PFC) is involved in bias perception by looming sounds. In a human electroencephalography study, neural signals in PFC differentiated looming and receding stimuli at an early stage, indicating a faster top–down control on prioritizing approaching events *via* PFC (Bidelman and Myers, 2020). Its involvement in regulating PPI and attentional modulation of PPI has also been well documented. In rats, a behavioral positron emission tomography study reported that the left frontal cortex (area 3) and the left and right prelimbic cortex were related to PPI modulation (Rohleder et al., 2016). Furthermore, a relationship between decreased function and volume of the medial PFC and low PPI values was found (Tapias-Espinosa et al., 2019). It has been further suggested that the PFC mediates the attentional enhancement of PPI of the acoustic startle reflex (Meng et al., 2020). Additionally, prefrontal dysfunction caused by isolation stress during adolescence resulted in PPI deficits (Drzewiecki et al., 2021; Du et al., 2021). What other brain areas are involved in and how the brain network subserving attention is involved in this process need further investigation.

Isolation rearing abolished the looming sound–induced PPI enhancements to some extent. We found that in socially reared rats, looming sounds caused higher PPI than stationary and receding sounds with the same duration. Although isolation rearing did not abolish their discrimination ability for looming and stationary sounds, it impaired their discrimination between looming and receding sounds as no difference of PPI was found. The failure of differentiating looming and receding sounds in isolation-reared rats may be related to their flawed attention (Li et al., 2009; Wu et al., 2018). Previously we found that isolation rearing abolished both fear conditioning–induced PPI enhancement (Du et al., 2010; Wu et al., 2016) and perceptual spatial separation–induced PPI enhancement (Du et al., 2009; Wu et al., 2016), which indicates an impairment of attending to the prepulse with acquired salient valence. Our study further extends it to show that isolation rearing abolished their attention to intrinsic salient value of prepulse (i.e., looming sounds) without acquisition. From evolutionary perspective, the failure of attending to potential biological salience of looming cues and lack of adaptive bias for an approaching object may leave their life at risk in the real world. PPI is a cross-species paradigm, which has been commonly used in animals (Wilkinson et al., 1994; Li et al., 1998a,b, 2009; Weiss et al., 2000; Swerdlow et al., 2001; van den Buuse et al., 2003; Zou et al., 2007; Du et al., 2009, 2011; Li, 2009; Uehara et al., 2009; Zhao et al., 2013; Lei et al., 2014; Wu et al., 2016, 2019; Ding et al., 2019) and humans (Li et al., 2009; Lei et al., 2018). The looming effect–induced attentional modulation of PPI can be used as a behavioral paradigm to probe attentional deficits in animal models and patients with mental disorders, such as schizophrenia (Dawson et al., 1993; Chen and Faraone, 2000; Dondé et al., 2020) and ADHD (Sharma and Couture, 2014; Tarver et al., 2014; Luo et al., 2019).

There are some limitations to our current study. First, the chosen frequency and intensity of looming and receding prepulse stimuli may make rats not to detect all the three components of the harmonic tone complex. To make a comparison to external attentional modulation of PPI paradigms (Zou et al., 2007; Du et al., 2011; Wu et al., 2016), we chose the same broadband noise at 60 dB SPL as background noise masker and the three-harmonic tone complex (1.3, 2.6, 3.9 kHz) as prepulse stimuli in our study. This was not an issue for stationary prepulse stimuli (67 dB SPL) but caused the intensity of the looming and receding prepulse (17–67 dB SPL) below background noise for some time (approximately 40 ms when the intensity was below 25 dB SPL, see Figure 1) and thus it is hard for rats to detect 1.3 and 2.6 kHz of the three-harmonic tone complex. Additionally, our rats may not be able to detect the 1.3 and 2.6 kHz tone of the three-harmonic tone complex at 17 dB SPL, since their hearing threshold for 1–2 kHz tones is approximately 25 dB SPL (Kelly and Masterton, 1976; Borg, 1982; Heffner et al., 1994). As the hearing threshold for 3–4 kHz tones is approximately 15 dB SPL (Kelly and Masterton, 1976; Borg, 1982; Heffner et al., 1994), our rats could detect 3.9 kHz of the three-harmonic tone complex for both looming and receding prepulse stimuli (17–67 dB SPL). Second, the lower detection of 1.3 and 2.6 kHz tone of the three-harmonic tone complex may cause the interval between prepulse and startling stimuli differed for looming and receding prepulse conditions. As stated above, the two tones were most likely detected for approximately 80 ms in a 120 ms prepulse. Consequently, the time between the recognized prepulse and the startle stimulus was longer in the receding prepulse condition than in the looming prepulse condition. This effect was additionally increased as the background noise was 60 dB SPL during each session and may affect the effectiveness of the prepulse on the PPI of the acoustic startle response. Third, the rats tested in Experiment 1 were involved in another attentional modulation of PPI paradigms, in which they have encountered a 50-ms three-harmonic tone complex (1.3, 2.6, 3.9 kHz) and another three-harmonic tone complex (2.3, 4.6, and 6.9 kHz). As the previous prepulse had the same frequency range of the one used in current study, their early exposure to the same frequency prepulse may cause less sensitivity to the looming prepulse used in our study and therefore their PPI was lower compared with the PPI in socially reared rats in Experiment 2.

In conclusion, our study showed a looming effect–induced PPI enhancement, which is that rising-intensity sound at an adequate time duration could effectively dampen the startle response elicited by a sudden stimulus, therefore it helps organisms to adapt to the complex environment by recruiting attentional and physiological resources. Isolation rearing impaired the abilities of the animals to discriminate between the looming and receding sounds, which makes them vulnerable to the approaching predators in the wild.

## Data Availability Statement

The raw data supporting the conclusions of this article will be made available by the authors, without undue reservation.

## Ethics Statement

The animal study was reviewed and approved by the Beijing Laboratory Animal Center, in the School of Psychological and Cognitive Sciences at Peking University.

## Author Contributions

ZW, XB, LeL, and LiL conceived the project. LiL supervised this research project. ZW and XB conducted the experiment. ZW performed the data analysis. ZW and LiL interpreted the data and wrote the manuscript. All authors contributed to the article and approved the submitted version.

## Conflict of Interest

The authors declare that the research was conducted in the absence of any commercial or financial relationships that could be construed as a potential conflict of interest.

## Publisher’s Note

All claims expressed in this article are solely those of the authors and do not necessarily represent those of their affiliated organizations, or those of the publisher, the editors and the reviewers. Any product that may be evaluated in this article, or claim that may be made by its manufacturer, is not guaranteed or endorsed by the publisher.
